# Influence of a Pediatric Fruit and Vegetable Prescription Program on Child Dietary Patterns and Food Security

**DOI:** 10.3390/nu13082619

**Published:** 2021-07-29

**Authors:** Amy Saxe-Custack, Jenny LaChance, Jennifer Jess, Mona Hanna-Attisha

**Affiliations:** 1 Department of Food Science and Human Nutrition, Division of Public Health, Michigan State University–Hurley Children’s Hospital Pediatric Public Health Initiative, 200 E 1st St, Flint, MI 48502, USA; 2 Division of Public Health, Michigan State University–Hurley Children’s Hospital Pediatric Public Health Initiative, 200 E 1st St, Flint, MI 48502, USA; jlachan1@hurleymc.com; 3 Department of Pediatrics and Human Development, Michigan State University College of Human Medicine, 200 E 1st St, Flint, MI 48502, USA; jessjenn@msu.edu; 4 Department of Pediatrics and Human Development, Division of Public Health, Michigan State University–Hurley Children’s Hospital Pediatric Public Health Initiative, 200 E 1st St, Flint, MI 48502, USA; hannamon@msu.edu

**Keywords:** fruit and vegetable prescriptions, food security, children, nutrition

## Abstract

Limited access to fresh foods is a barrier to adequate consumption of fruits and vegetables among youth, particularly in low-income communities. The current study sought to examine preliminary effectiveness of a fruit and vegetable prescription program (FVPP), which provided one USD 15 prescription to pediatric patients during office visits. The central hypothesis was that exposure to this FVPP is associated with improvements in dietary patterns and food security. This non-controlled longitudinal intervention trial included a sample of caregiver–child dyads at one urban pediatric clinic who were exposed to the FVPP for 1 year. Patients received one USD 15 prescription for fresh produce during appointments. A consecutive sample of caregivers whose children were 8–18 years of age were invited to participate in the study. Dyads separately completed surveys that evaluated food security and dietary behaviors prior to receipt of their first prescription and again at 12 months. A total of 122 dyads completed surveys at baseline and 12-month follow-up. Approximately half of youth were female (52%), and most were African American (63%). Mean caregiver-reported household food security improved from baseline to 12 months (*p* < 0.001), as did mean child-reported food security (*p* = 0.01). Additionally, child-reported intake of vegetables (*p* = 0.001), whole grains (*p* = 0.001), fiber (*p* = 0.008), and dairy (*p* < 0.001) improved after 12 months of exposure to the FVPP. This study provides evidence that pediatric FVPPs may positively influence food security and the dietary patterns of children.

## 1. Introduction

Lifelong dietary patterns are established during childhood and adolescence [1,2,3]. Influenced by various factors, including caregiver and peer eating behaviors as well as food attitudes, costs, and availability [4,5,6], consumption patterns during this critical period also play a key role in the development of certain chronic health conditions [3,7,8,9]. Regrettably, most US children and adolescents fail to consume adequate amounts of high-nutrient foods, particularly fruits and vegetables [10,11,12,13], which provide important protections against chronic disease [8,9,14,15,16]. Persistent environmental challenges to achieving dietary recommendations among youth, particularly in low-income communities, include limited access to and affordability of high-quality, fresh foods [17,18,19,20,21].

Recent efforts to simultaneously address food insecurity and poor dietary behaviors among children and adolescents include the introduction of pediatric fruit and vegetable prescription programs. These programs vary widely in design and approach; however, most involve physician-issued prescriptions that may be exchanged for fresh fruits and vegetables at local farmers’ markets, mobile markets, or food stores. Although evidence suggests that prescriptions for fruits and vegetables may address barriers to healthy food access among young patients and their families [22,23,24,25], reproducible implementation strategies that consistently demonstrate effectiveness are absent.

In August 2015, a large university-affiliated pediatric office, in a low-income, urban area, moved to a downtown farmers’ market building. Shortly after this move, the clinic developed and implemented a successful pediatric fruit and vegetable prescription program (FVPP) that provided a pediatrician-issued prescription for fresh produce to patients (birth to 18 years of age) at every office visit [22,26]. This FVPP was expanded to a second pediatric clinic located several miles from the downtown farmers’ market in August 2018. The current study sought to examine the preliminary effectiveness of the expanded FVPP, which provided one USD 15 prescription to all pediatric patients at the conclusion of office visits. Prescriptions were redeemable only for fresh produce at either the downtown farmers’ market or local mobile market.

## 2. Materials and Methods

### 2.1. Study Population

Flint, Michigan, the birthplace of General Motors, is home to approximately 100,000 residents. Following the American automobile industry’s decline, the city fell into an extreme recession [27]. The child poverty rate in Flint is nearly 60% [28], and the city lacks resources and dietary options. Local stores are likely to offer low-quality foods and few healthy food options [26,29,30], and grocery stores are limited within the city [31].

### 2.2. Pediatric Fruit and Vegetable Prescription Program

Hurley Children’s Center, a residency-training clinic with approximately 11,000 visits annually, launched Michigan’s first pediatric FVPP in February 2016. The program was intentionally designed to facilitate ease of implementation within busy pediatric offices while sending a ubiquitous message to patients and families regarding the importance of regular consumption of fruits and vegetables. Prescriptions for fresh fruits and vegetables were built into the existing electronic medical record (EMR) system and stored in patient records. This allowed for ease of distribution as well as monthly tracking of prescription distribution rates. Pediatricians ordered prescriptions through the EMR system, printed on prescription paper, and distributed to all patients (birth to 18 years of age) at the conclusion of office visits.

Following the success of the FVPP at Hurley Children’s Center [22,26], an identical program was introduced in August 2018 at a private practice pediatric clinic in Flint. This second clinic, Akpinar Children’s Clinic, serves approximately 3000 patients. Most patients are residents of Flint and receive public health insurance. Modeled after the original program, fruit and vegetable prescriptions at Akpinar Children’s Clinic were ordered via EMR, printed, and given to patients (birth to 18 years of age). All patients, regardless of income or health status, received one USD 15 fruit and vegetable prescription at every clinic visit to be exchanged for fresh produce at either the year-round, downtown farmers’ market or a mobile market that also offered free delivery of fresh produce boxes. Prescriptions were treated as vouchers redeemable only for fresh fruits and vegetables and were valid for 90 days from prescription receipt.

### 2.3. Study Design

This was a non-controlled longitudinal intervention trial with a consecutive sample of 122 caregiver–child dyads exposed to the pediatric FVPP for 1 year. Dyads completed in-person assessments at baseline and approximately 6-month and 12-month follow-up with a trained research assistant. Descriptions of the evaluation tools are also available in an earlier article reporting baseline data and a 6-month follow-up on fruit intake [23,32]. The current study was approved by Michigan State University’s Institutional Review Board.

### 2.4. Participants and Data Collection

Beginning in August 2018, pediatric patients at Akpinar Children’s Clinic received one USD 15 prescription for fresh produce during each office visit. A consecutive sample of caregivers whose children were between 8 and 18 years of age was invited to participate in this study. Exclusion criteria included the following: caregiver or child not English speaking, legal guardian not present at enrollment, child assent refused, or sibling previously enrolled (study enrollment was limited to 1 caregiver and 1 child per household).

Caregiver–child dyads provided consent and assent before separately answering demographic questions and survey questions that evaluated food security and dietary behaviors. Approximately 12 months after baseline data collection, caregivers returned to the clinic with their children to complete follow-up surveys. All data were collected from August 2018 through March 2020 using a secure digital platform (Michigan State University Qualtrics), accessed through iPads.

### 2.5. Evaluation Tools

#### 2.5.1. Food Security

To measure household food insecurity and hunger, caregivers completed the National Center for Health Statistics’ US Household Food Security Module: Six Item Short Form [33]. Food security status can be understood using the household’s raw score (0–1 = high/marginal food security; 2–4 = low food security; 5–6 = very low food security), calculated by counting affirmative responses (“often”, “sometimes”, “yes”, “almost every month”, “some months but not every month”).

Children 12 years of age and older (*n* = 67) completed the 9-question Self-Administered Food Security Survey Module for Youth. Because the module’s internal validity is adequate for children ages 12 years and older but is not recommended for younger children, the tool was not used with children younger than 12 years of age [34]. The sum of affirmative responses (“a lot” or “sometimes”) served as the child’s raw score. Food security status can be understood using the raw score (0–1 = high/marginal food security; 2–5 = low food security; 6–9 = very low food security).

#### 2.5.2. Dietary Behaviors of Children

The Block Kids Food Screener (BKFS), a 41-item food frequency questionnaire with relatively low administration burden, assessed usual and long-term eating behaviors. Prior research demonstrates it has good relative validity for children and adolescents [35]. The BKFS documented the frequency and quantity of foods and beverages consumed during the previous week and was completed by children with the help of a trained research assistant. Dietary analysis, using the Block Online Analysis System, produced nutrient estimates and number of servings by food group.

#### 2.5.3. Statistical Analyses

Demographic data were analyzed using descriptive statistics, specifically means with standard deviations and frequencies with percentages. To compare change at 12 months from baseline, a series of paired t-tests assessed change in mean daily intake of key food groups as well as child-reported food security and household food security. Additionally, independent t-tests examined whether mean change in daily intake of vegetables, fruits, fiber, whole grains, and dairy differed by key child demographics (age group, gender, and race/ethnicity). Change in daily intake of vegetables, fruits, fiber, whole grains, and dairy was calculated by subtracting each value at baseline from the daily intake value at 12 months. McNemar analysis was used to determine whether there was a significant increase in the number of children who reported daily consumption of at least ¼ cup, ½ cup, and 1 cup of vegetables and fruits at the 12-month follow-up. Finally, multiple logistic regression examined the relationship between key child demographics and those who reported an increase of at least ¼ cup of vegetables and fruits at the 12-month follow-up. In relation to logistic regression analyses, for age group, younger age was coded as 1; for gender, male was coded as 1; and for race/ethnicity, African American was coded as 1. Data were analyzed using IBM SPSS Statistics version 27.

## 3. Results

### 3.1. Demographics

A total of 122 caregiver–child dyads (244 participants) completed surveys at baseline and 12-month follow-up, with the majority (70%) reporting residency in Flint. As shown in Table 1, approximately half of youth were female (52%), and most were African American (63%). Age of children ranged from 8 to 18 years (mean age 12.42 ± 2.78). Most caregivers were female (93%) and African American (59%). Thirty-seven percent of caregivers reported having a high school degree or less.

### 3.2. Distribution Rate

From August 2018 through September 2019, a total of 7827 patients (birth to 18 years of age) visited Akpinar Children’s Clinic, and 5953 prescriptions for fresh fruits and vegetables were ordered via EMR and distributed to patients. This reflects a 76% prescription distribution rate during the program’s inaugural year.

### 3.3. Food Security

The US Household Food Security Module was completed by 122 caregivers at baseline and 12-month follow-up. Mean household food security score decreased significantly (*p* < 0.001) from baseline (1.96 ± 2.20) to the 12-month follow-up (0.87 ± 1.25), indicating an improvement in food security. There was no difference in change in caregiver-reported household food security score by caregiver age group (*p* = 0.47), caregiver-reported race/ethnicity (*p* = 0.85), or caregiver education level (*p* = 0.45). A total of 67 children (≥12 years of age) completed the Food Security Survey Module for Youth. Mean child-reported food security score also decreased significantly (*p* = 0.01) from baseline (1.88 ± 2.06) to 12-month follow-up (1.04 ± 1.97), indicating an improvement in food security. There was no difference in change in child-reported food security score by child age group (*p* = 0.55), gender (*p* = 0.85), or between those who reported race/ethnicity as African American or white (*p* = 0.12).

### 3.4. Dietary Behaviors of Children

The BKFS was completed at baseline and 12-month follow-up by 122 children. As shown in Table 2, children reported improvements in mean daily intake of vegetables (*p* = 0.001), whole grains (*p* = 0.001), fiber (*p* = 0.008), and dairy (*p* < 0.001) after 12 months of exposure to the FVPP. There were no significant differences in change in mean daily intake of vegetables, whole grains, fiber, or dairy by child age group, child gender, or child race/ethnicity (Table 3). Although change in mean daily intake of total fruit in the entire sample of children was not significant, there was a significant difference in mean change of daily fruit intake when comparing consumption of total fruits by race/ethnicity, with African American children reporting a mean decrease of 0.3201 cups of fruit per day and white children reporting a mean increase of 0.209 cups of fruit per day (*p* = 0.04). There was no difference by child age group or gender in reported intake of total fruit (Table 3).

Given the program’s focus on improving intake of fruits and vegetables, an examination of the percentage of children who achieved dietary recommendations for mean daily intake of fruits and vegetables was considered. Unfortunately, very few children met current dietary recommendations for fruit and vegetable intake at either timepoint. As a result, we examined the number of children who reported mean daily consumption of at least ¼ cup, ½ cup, and 1 cup of vegetables and fruits at baseline and 12-month follow-up (Table 4). There was a significant increase in the number of children who reported daily consumption of at least ¼ cup (*p* < 0.001), ½ cup (*p* < 0.001), and 1 cup (*p* = 0.001) of vegetables at the 12-month follow-up. Additionally, there was a significant increase in the number of children who reported daily intake of at least ½ cup (*p* = 0.02) and 1 cup (*p* = 0.003) of total fruits at the 12-month follow-up.

Using a logistic regression, the impact of child race/ethnicity, gender, and age group on the increase of at least a ¼ cup in intake of fruits and vegetables was examined. For vegetables, the model was significant (Χ^2^ = 8.14, *p* = 0.04), indicating that these demographic variables were predictive of who reported an increase of at least ¼ cup intake of vegetables at 12 months. In this model, age group (*p* = 0.06, 95% CI 0.97–4.87) and gender (*p* = 0.51, 95% CI 0.59–2.87) were not significant, while race/ethnicity was significant. Children who reported their race/ethnicity as African American were less likely (Exp (B) = 0.39, 95% CI 0.16–0.93) to have increased their daily intake of vegetables by ¼ cup at the 12-month visit when compared to children who reported their race/ethnicity as white (*p* = 0.03). For total fruits, the model was also significant (Χ^2^ = 10.92, *p* = 0.01). Here, age group (*p* = 0.43, 95% CI 0.61–3.23) was not significant, while gender (Exp (B) = 0.36, 95% CI 0.15–0.82, *p* = 0.02) and race/ethnicity (Exp (B) = 0.37, 95% CI 0.15–0.88, *p* = 0.03) were significant. Male children and those children who reported their race/ethnicity as African American were less likely to have increased their daily intake of fruit by at least ¼ cup at 12 months when compared to female children and those children who reported their race/ethnicity as white, respectively.

## 4. Discussion

Impacting approximately 6.5 million US children, food insecurity is associated with serious consequences that include poor diet quality [36,37,38], negative health and behavioral outcomes [39,40,41,42], and low academic achievement [41,43]. Even intermittent food insecurity, which causes occasional or modest undernutrition among youth, is likely to have long-term neurocognitive and developmental implications [41,44,45,46]. Pediatricians have a responsibility to screen households with children for food insecurity [47], but resources to address the underlying issues are frequently lacking [48,49]. Previous research has suggested that caregivers whose children were exposed to the pediatric FVPP at Akpinar Children’s Clinic perceived the program to be effective in improving household food security, with many describing how they held onto prescriptions to redeem for fruits and vegetables when food resources were depleted [26]. Consistent with this qualitative finding, caregivers and children in the current study reported significant improvements in measured food security following 1 year of exposure to the identical FVPP. Implemented within two distinctly different clinic environments, the FVPP successfully addressed not only food insecurity, but also nutrition security, specifically the provision of healthy foods.

Unique in design, the current FVPP provided prescriptions for fruits and vegetables to all pediatric patients, regardless of health condition or socioeconomic status, to emphasize the important role fruits and vegetables have in health promotion and disease prevention among all children [1,2,3]. This primary prevention approach is markedly different from previous efforts that have employed produce prescriptions for adults with diet-related chronic health conditions as a disease-management strategy [50,51,52,53,54]. With fruit and vegetable consumption continually falling short of national goals among US children and adolescents [13,55,56,57], the current program was intentionally designed to send a consistent and straightforward message regarding the importance of fruits and vegetables in a healthy diet. Pediatricians actively promoted that message through the provision of prescriptions that enabled children to purchase fresh, high-quality fruits and vegetables. Previous research has indicated that simply giving fruits and vegetables to children is likely to have an important influence on dietary intake through familiarization [21]. Repeated exposure is, in fact, a key mechanism through which youth food acceptance occurs [58,59]. Although participants in the current study failed to meet current dietary recommendations, noteworthy improvements in mean daily consumption of vegetables, whole grain, fiber, and dairy were consistently reported across all age, gender, and ethnic categories. Because greater fruit and vegetable intake during childhood is associated with reductions in chronic diseases in adulthood [8,9,14,15,16], this particular finding, which is consistent with previous research [23,25,26], highlights the potential long-term implications of pediatric prescriptions for fruits and vegetables.

Although exposure to the FVPP was associated with important dietary improvements among all participants, change in mean daily intake of total fruits differed by race/ethnicity. African American children reported a decrease in total fruit consumption over 12 months, while white children reported an increase in consumption. Further analysis also suggested that, when compared to white children, African American youth were less likely to increase the intake of fruits and vegetables by at least ¼ cup. These findings support previous research that has noted similar differences in consumption by race/ethnicity [11,60]. The current FVPP may benefit from tailored nutrition education that carefully considers racial/ethnic factors that may influence dietary choices among youth. Additionally, results highlight the continued need to actively address health disparities when implementing similar nutrition interventions.

Recent evidence illustrates parental desire for healthcare systems to not only focus on interventions at the clinic and community levels, but to also advocate for more expansive policies for alleviating barriers to healthy foods [61]. Pediatric prescriptions for fruits and vegetables are increasingly demonstrating effectiveness in combatting food insecurity [24,26] and poor dietary patterns [23,25,26] among children. The current study provides evidence of an effective and reproducible model for widespread prescription distribution within various clinic settings. Although the current initiative was supported by foundation funding through a competitive grants process, opportunities for federal grant support are emerging. Produce prescription programs were added to the US Farm Bill in 2018 through the Gus Schumacher Nutrition Incentive Program. This program allocated USD 25 million toward FVPPs and committed to increasing funding to USD 56 million by 2023 [62].

Limitations of the current study include the lack of a control group. However, pediatricians and community partners questioned whether the inclusion of a control group was ethical when children in Flint are facing hunger and food insecurity. The sample was small and specific to one low-income, urban community. As a result, findings may not be generalizable. However, the current fruit and vegetable prescription program could be modeled in similar communities confronted with enduring obstacles to healthy food access and affordability. Additionally, there may have been selection bias as responses from dyads who chose not to participate may have differed from those who voluntarily enrolled. However, characteristics of the study population closely match those of the source population of predominantly low-income, minority families receiving public health insurance. Finally, the accuracy of the BKFS may be limited by recall bias, but a trained research assistant was consistently available when children completed this instrument to minimize this limitation.

## 5. Conclusions

Pediatricians have a primary role in not only identifying children who are at risk for food insecurity or poor diet and connecting them to community resources, but also in advocating for policies that support access to healthy foods for children of all income levels [47]. The current study provides evidence that fruit and vegetable prescriptions, easily ordered through EMR systems and provided to all pediatric patients, may have a significant influence on food insecurity and dietary patterns of children living in a low-income, urban community. In addition to monitoring the long-term impact of the FVPP, future research will investigate the influence of prescription distribution and redemption patterns on food security and dietary behaviors of children.

## Figures and Tables

**Table 1:** Child and caregiver demographics.

Demographics		Number	Percentage
Dyad residency	Flint	86	70
	Not Flint	36	30
Child gender	Male	58	48
	Female	64	52
Child-reported race/ethnicity	African American	77	63
	White	33	27
	Other/Not reported	12	10
Child age (mean age 12.42 ± 2.78)	8–12 years	67	55
	13–18 years	55	45
Caregiver gender	Male	8	7
	Female	114	93
Caregiver-reported race/ethnicity	African American	72	59
	White	35	29
	Other/Not reported	15	12
Caregiver age group (mean age 39.94 ± 10.28)	25–34 years	43	36
	35–44 years	46	38
	45 or more years	32	26
Caregiver education	High school degree or less	44	37
	Some college/technical degree	51	43
	Bachelor’s degree or more	23	19

**Table 2:** Child-reported mean daily intake.

Food Group	Timing	Mean ± SD	*p* Value
Vegetables (cups)	Baseline	0.68 ± 0.62	0.001
	12 Months	0.91 ± 0.43	
Total fruits (cups)	Baseline	1.36 ± 1.19	0.39
	12 Months	1.26 ± 0.64	
Fiber (grams)	Baseline	10.11 ± 6.76	0.008
	12 Months	12.10 ± 4.92	
Whole grains (ounces)	Baseline	0.49 ± 0.52	0.001
	12 Months	0.69 ± 0.49	
Dairy (cups)	Baseline	1.34 ± 1.03	<0.001
	12 Months	1.79 ± 0.95	

**Table 3:** Comparison of change in key dietary intake by child demographics.

Child Demographics		Vegetables		Total Fruit		Fiber		Whole Grains		Dairy	
		Change	*p*	Change	*p*	Change	*p*	Change	*p*	Change	*p*
Gender	Male	0.23 ± 0.73	0.88	−0.30 ± 1.26	0.08	1.98 ± 8.47	0.99	0.19 ± 0.75	0.83	0.46 ± 1.44	0.91
	Female	0.21 ± 0.70		0.09 ± 1.20		2.01 ± 7.90		0.22 ± 0.63		0.43 ± 1.11	
Race/ethnicity	African American	0.11 ± 0.76	0.06	−0.32 ± 1.34	0.04	0.48 ± 8.46	0.05	0.14 ± 0.76	0.25	0.42 ± 1.35	0.77
	White	0.39 ± 0.58		0.21 ± 0.98		3.81 ± 7.30		0.31 ± 0.60		0.34 ± 1.17	
Age group	8–12 years	0.25 ± 0.72	0.59	0.02 ± 1.07	0.27	2.70 ± 7.74	0.29	0.19 ± 0.70	0.84	0.44 ± 1.29	0.98
	13–18 years	0.18 + 0.71		−0.24 ± 1.41		1.14 ± 8.58		0.22 ± 0.69		0.45 ± 1.27	

**Table 4:** Change in number of children reporting vegetable and fruit intake of at least ¼, ½, and 1 cup.

Food Group	Timing	At Least ¼ Cup		At Least ½ Cup		At Least 1 Cup	
		*n* (%)	*p* Value	*n* (%)	*p* Value	*n* (%)	*p* Value
Vegetables	Baseline	97 (80%)	<0.001	62 (51%)	<0.001	26 (21%)	0.001
	12 Months	116 (95%)		103 (84%)		50 (41%)	
Total Fruits	Baseline	107 (88%)	0.10	93 (76%)	0.02	63 (52%)	0.003
	12 Months	115 (94%)		107 (88%)		83 (68%)	

## Data Availability

Requested data may be provided after IRB approval and appropriate data use agreements have been obtained.
